# Nociceptin Receptor Is Overexpressed in Non-small Cell Lung Cancer and Predicts Poor Prognosis

**DOI:** 10.3389/fonc.2019.00235

**Published:** 2019-04-05

**Authors:** Kaiyuan Wang, Yu Zheng, Yinli Yang, Jian Wang, Baihui Li, Feng Wei, Hongwei Zhao, Xiubao Ren

**Affiliations:** ^1^Key Laboratory of Cancer Prevention and Therapy of Tianjin, Department of Immunology, National Clinical Research Center for Cancer, Tianjin's Clinical Research Center for Cancer, Tianjin Medical University Cancer Institute and Hospital, Tianjin, China; ^2^Key Laboratory of Cancer Prevention and Therapy of Tianjin, Department of Anesthesiology, National Clinical Research Center for Cancer, Tianjin's Clinical Research Center for Cancer, Tianjin Medical University Cancer Institute and Hospital, Tianjin, China; ^3^Key Laboratory of Cancer Immunology and Biotherapy, Tianjin, China; ^4^Key Laboratory of Cancer Prevention and Therapy of Tianjin, Department of Integrative Oncology, National Clinical Research Center for Cancer, Tianjin's Clinical Research Center for Cancer, Tianjin Medical University Cancer Institute and Hospital, Tianjin, China; ^5^Key Laboratory of Cancer Prevention and Therapy of Tianjin, Department of Biotherapy, National Clinical Research Centre for Cancer, Tianjin's Clinical Research Center for Cancer, Tianjin Medical University Cancer Institute and Hospital, Tianjin, China

**Keywords:** receptor, nociceptin, carcinoma, non-small-cell lung, receptors, opioid, morphine, survival analysis

## Abstract

Classic opioid receptors, mu (μ), delta (δ), and kappa (κ), have been reported to be expressed in non-small cell lung cancer (NSCLC) cell lines and tumor tissues and to play a role in tumor prognosis. However, the expression and role of the non-classic opioid receptor, nociceptin receptor (NOP) in cancer are unclear. Our hypothesis was that NOP was also highly expressed in NSCLC tumor tissues and this could be correlated with patients' prognostic characters. Expression of NOP was examined in archived cancer tissues from 129 enrolled NSCLC patients by immunohistochemistry and was further analyzed with the patients' outcomes. NOP expression in NSCLC cell lines was also detected. The dataset from Kaplan-Meier Plotter was used to explore the correlation between the levels of NOP mRNA in cancerous tissue and the prognosis of NSCLC patients. Cell functional assays were performed to detect the effect of NOP activation on tumor aggressive furthers. Results showed NOP expression was highly expressed in cancer tissues and human cancer cell lines. NOP expression was not associated with patients' opioid requirement but closely with some clinicopathological indicators which reflected the malignancy. Moreover, NOP staining level was the independent poor prognostic factor for NSCLC patients receiving lobectomy, which was further verified by determining the mRNA expression levels through the online dataset. *In vitro* experiments revealed that NOP activation promotes the proliferation and invasion of A549 cells via PI3K/Akt signaling pathway. We conclude that NOP is overexpressed in NSCLC and is inversely correlated with patient's postoperative survival.

## Introduction

Advances have recently been achieved in targeted therapies and immunotherapies for lung cancer; however, it remains the leading cause of cancer-related death in both men and women worldwide, with an unacceptably low 5-year survival rate, even for early-stage cancer patients (1, 2). Non-small cell lung cancer (NSCLC) is the most common type of lung cancer, accounting for 80–85% of all lung cancer diagnoses. Thus, efforts for early diagnosis of NSCLC, especially by identifying potential tumorigenic targets, and developing novel therapeutic strategies are urgently required.

For NSCLC patients receiving lobectomy or cancer pain treatment, opioids such as morphine, hydromorphone, fentanyl, and oxycodone are the most widely used and effective analgesics (3). Their receptors, mainly classic opioid receptors including mu (μ), delta (δ), and kappa (κ) (MOR, DOR, and KOR) have been reported to be expressed in NSCLC cell lines or in tumor tissues, and they perform different functions correlated with the clinical prognosis. For example, MOR and DOR are both upregulated in NSCLC cell lines as well as in the lung cancer tissue samples (4, 5). Moreover, increased expression of MOR is associated with progression of NSCLC and the activation of MOR leads to tumor growth by coactivating epidermal growth factor receptor (EGFR) pathways (6). KOR, however, stimulated by its ligand or agonist, could inhibit the proliferation of NSCLC through phosphorylated-glycogen synthase kinase 3b (p-GSK3b) signaling pathway (7).

Nociceptin receptor (NOP) is identified as the fourth opioid receptor with a later recognized endogenous ligand nociceptin/orphanin FQ (8). It is a G protein-coupled receptor (GPCR) with seven transmembrane domains, and it shows 50% homology with the other three opioid receptors (9, 10). However, NOP activation exhibited a distinct biological effect with respect to pain, learning, memory, neuroendocrine control, emotional states, food intake, and motor control (11–13). Thus, NOP was generally characterized as a non-classical opioid receptor. NOP is highly expressed in the nervous system, including the brain, spinal cord, and dorsal root ganglion (DRG) neurons, as well as in the immune cells (14, 15). In advanced cancer patients, NOP expression in peripheral blood cells was higher than in healthy controls (16). However, the expression of NOP in NSCLC tumor cell lines as well as in cancer tissues has not yet been studied.

The present study aimed to investigate the expression and role of NOP in NSCLC cell lines and cancer patients. The hypothesis that NOP is highly expressed in NSCLC tissues, with potential correlation with the clinicopathological data and the outcome of NSCLC patients, was tested in this study.

## Materials and Methods

### Patients

#### Inclusion and Exclusion Criteria

This study was approved by the Ethical Committee of Tianjin Medical University Cancer Institute and Hospital (TMUCIH), and a written, informed consent was obtained from each participant or their legal custodian, in accordance with the Declaration of Helsinki. A total of 129 lung cancer patients who had undergone pulmonary resection and systemic lymph node dissection in TMUCIH from April 2008 to August 2014 were enrolled in the study. All patients had been pathologically diagnosed for NSCLC for the first time when enrolling and the TNM stages were in accordance with the latest International Association for the Study of Lung Cancer TNM classification system (17). The postoperative treatment, including adjuvant chemotherapy, radiotherapy or targeted therapy, was strictly ground on the National Comprehensive Cancer Network Clinical Practice Guideline in NSCLC. Exclusion criteria included patients with pre-existing or simultaneous tumors; patients receiving preoperative anticancer treatment such as radiotherapy, neo-adjuvant chemotherapy, or immunotherapy, and patients who died within 1 month after surgery.

All participants underwent 18F-fluorodeoxyglucose positron emission tomography combined with computed tomography (18F-FDG-PET/CT) before surgery, and the maximum standardized uptake value (SUVmax) was obtained.

Each enrolled patient had an integral clinicopathological and follow-up record. All patients were followed up until August 30, 2016. Patients who were still alive at the last follow-up were censored in the present study. Overall survival (OS) was defined as the time from surgery to death by any cause or to the last follow-up. Progression-free survival (PFS) was defined as the interval between surgery and the diagnosis of recurrence or the last date of follow-up.

#### Opioid Requirement

The opioid requirements of all participants from diagnosis to death or the last follow-up were also recorded. The opioid prescriptions were converted into oral morphine equivalents (OME) using the equi-analgesic conversion tables and further presented as daily dosage (18). The amount of 5 mg/day OME was set as the cut-off pursuant to the previous literature for it could differentiate short-term or occasional opioid use from long-term opioid schedule in NSCLC patients (19). The enrolled patients were then divided into high opioid requirement group and low opioid requirement accordingly.

### Immunohistochemistry

The primary antibody of NOP used for immunohistochemistry (IHC) of lung carcinoma tissues was a rabbit polyclonal NOP antibody obtained from Santa Cruz Biotechnology (Santa Cruz, Dallas, TX, cat No. sc15309) with final dilution 1:100. To compare the expression levels of NOP in normal lung tissue with those in cancer tissue, 60 samples of normal lung tissue located more than 10 cm away from the tumor were taken as para-carcinoma controls. Paraffin-embedded specimens were prepared and archived at the Department of Pathology, TMUCIH. Specimens were baked for 1 h at 70°C for dewaxing. Samples were then fixed with formaldehyde; antigen retrieval was performed using heat mediation with citrate buffer (10 mM, pH 6.0). Endogenous peroxidase activity was blocked with 0.3% hydrogen peroxide for 20 min. The specimens were then incubated overnight with the NOP primary antibody at the aforementioned dilution at 4°C. After rehydration with PBS, an undiluted horseradish peroxidase (HRP)-conjugated goat anti-rabbit IgG was added to the samples as the secondary antibody for 30 min at 37°C. The slides were rinsed and incubated with 3, 3′-Diaminobenzidine (DAB) chromogen substrate (ZSGB-BIO, Beijing, China). Nuclei were counterstained by haematoxylin (Sigma-Aldrich, Buchs, Switzerland). Finally, the tissue sections were sealed by mounting a glass coverslip and viewed using a light microscope.

Two pathologists who were blinded to the identity of the samples assessed the tumor and para-cancer sections and arrived at a consensus. NOP positive specimens were defined as those showing membranous or cytoplasmic staining of tumor cells. Briefly, IHC staining quantification was accomplished by the two-way scoring method that takes both staining intensity, and proportion of stained cells into account. The staining intensity was classified into four categories according to the color of immune reactions: negative, 0, no staining; weak, 1, light brown in color; moderate, 2, brown in color; and strong, 3, with dark brown staining. The proportion of positively stained cells was reported as: 0–25%, 1; 26–50%, 2; 51–75%, 3; and 76–100%, 4. The overall expression level of NOP was obtained by multiplying the staining intensity and the score of the proportion of positively stained cells. A median expression score of 6 was taken as the cut-off value, samples with scores of 0–4 were considered as low expressing, others with scores of 6–12 were defined as high expressing according to the published scoring system (20).

### Cell Culture

Different human lung cancer cell lines A549, H1299, H520, H460, and H446 and human lung bronchus epithelium cell line BEAS-2B were obtained from ATCC. They were cultured in RPMI medium 1640 (Gibco, Waltham, MA) supplemented with 10% fetal bovine serum (Hyclone, Logan, UT). Cells were then maintained in a humidified incubator equilibrated with 5% CO_2_ at 37°C.

### Tumor Tissue Samples

Fresh tumor and para-cancer tissues were obtained from 8 lung cancer patients receiving pulmonary resection in Department of Lung carcinoma, TMUCIH after receiving a written and informed consent. The utilization procedure of these tissues was approved by the Ethics Committee of TMUCIH. All patients had a pathology-based diagnosis of NSCLC before surgery and none of the patients had received radiotherapy, chemotherapy, or other medical intervention before sample collection.

### Quantitative Real-Time PCR (qRT-PCR)

Total RNA from cultured cancer cells was extracted using Trizol (Invitrogen, Carlsbad, CA) following the manufacturer's instructions. cDNA was synthesized by reverse transcription with the MMLV Reverse Transcriptase (Promega, Madison, WI). The sequences of primers were as following: NOP forward: 5′-TGCCGTTCTGGGAGGTTATCTA-3′ and reverse: 5′-TTAGGGTGAAGGTGCTGGTGA-3′. β-actin, forward 5-TGGCACCCAGCACAATGAA-3, and reverse 5′-CTAAGTCATAGTCCGCCTAGAAGCA-3′. Relative quantitation was performed with the ABI PRISM 7500 sequence detection system (Applied Biosystems, Foster City, CA) using the real-time SYBR green fluorescence PCR kit (Takara, Shiga, Japan) following the manufacturer's instructions. C_t_ values (cycle of threshold) were determined by the ABI software and the results were further analyzed by calculating the comparative Ct value (2^−ΔΔCt^) with β-actin as an internal control.

### Western Blot

The culture medium of the cancer cells was removed and the cells were washed twice with ice-cold PBS. The fresh tumor and para-cancer sample tissues were ground into a single-cell suspension. Harvested cells were then lysed in lysis buffer for 30 min at 4°C and centrifuged at 1,2000 × rpm for 10 min. The supernatants were collected and protein concentrations were measured using the BCA protein assay kit (Solarbio, Beijing, China) as per the manufacturer's instructions. Equal quantities of total protein were separated on a 10% SDS-PAGE gel and transferred onto polyvinylidene difluoride membranes (Millipore, Burlington, MA). The membranes were blocked in 5% non-fat milk for 1 h and then incubated overnight with rabbit polyclonal anti-NOP antibody (1:500, Abcam, Cambridge, MA, cat No. ab66219) and then with HRP-conjugated secondary antibodies for 1 h. The blots were developed using ECL Substrates (Amersham, Aldermaston, UK). GAPDH (Proteintech, Rosemont, IL) was used as an endogenous control for protein quantification.

### Cell Functional Experiments

#### Cell Viability Assay

Three thousand A549 cells were seeded in each well of a 96 well microplate with 100 μL cell culture media. Nociceptin (Tocris, Minneapolis, MN, cat No. 0910) with indicated concentration with or without UFP 101 (R &D, Minneapolis, MN, cat No. 1552) or Ly294002 (MedChem Express, Monmouth, NJ, cat No. HY-10108) were added into the well and co-incubated for 24, 48, and 72 h in a CO2 incubator. Ten microliter Cell Counting Kit-8 (CCK-8) reagent (Dojindo, Kumamoto, Japan) was added into each well and further incubated for 1–2 h. The absorbance was measured at 450 nm with a microplate reader. Each experiment was conducted 3 times.

### Two-Dimensional (2D) Clone Formation Assay

A549 cells were seeded in the 6-well plate (5,000 cells/well), and nociceptin with indicated concentration and UFP 101 or Ly294002 was added into the well for 72 h before the cells were stained with crystal violet. 3 wells of each group were counted for the number of cell clones, and each experiment was conducted 3 times.

#### Cell Invasion Assay

Transwell chamber filters (BD, San Jose, CA) were coated with Matrigel. After addition of nociceptin, A549 cells were suspended in serum-free media and then 2 × 10^4^ cells were seeded into the upper chamber in a volume of 500 μL. The chamber was then cultured in a well containing 500 μL of media with 10% fetal bovine serum at 37°C for 18 h. Cells on the upper side of the membrane were removed with cotton swabs and those on the other side were stained and counted. Four high-powered fields were counted for each membrane. Each experiment was conducted in triplicate.

### *In silico* Data Analysis

The dataset from Kaplan-Meier Plotter (http://kmplot.com) was used to explore the correlation between expression levels of NOP mRNA in cancerous tissue and the prognosis of NSCLC patients using a larger sample size. The probe used for analysis was 206564_at. By choosing auto select best cut-off, a total of 1926 lung cancer patients showing the histology of adenocarcinoma (ADC) and squamous cell carcinoma (SCC) were divided into NOP high expression group and NOP low expression group. The hazard ratio (HR) with 95% confidence intervals (CI) and the *P*-value of log-rank was calculated and significance level was set at *P* < 0.05.

### Statistics

SPSS 22.0 (IBM Corp, Armonk, NY) and GraphPad Prism 6.0 (GraphPad Software, San Diego, CA) were used to perform all the statistical analyses and to draw the figures. The Shapiro-Wilk test was used to assess the normality of data. NOP expression scores were presented as medians (inter-quartile range, IQR) and compared with the Mann-Whitney test. Fold changes in NOP mRNA transcription and quantification for Western blot, proliferation and invasion assays were presented as mean and standard error of mean (SEM), and compared using one-way ANOVA with Bonferroni's multiple comparisons test. Two-way ANOVA followed by Bonferroni's multiple comparisons test were applied to analyse dose and time dependent change of cell viability in CCK-8 assays. The categorical data were compared using Fisher's exact test or Pearson's chi-square test. The Kaplan-Meier method was applied to determine OS and PFS with log-rank test. Multivariable analysis of association between the independent factors and postoperative survival was performed by the Cox proportional hazard regression model. A two tailed *P* < 0.05 was considered statistically significant.

## Results

### NOP Expression in Human Lung Cancer Tissue and Cell Lines

**Figure 1:**
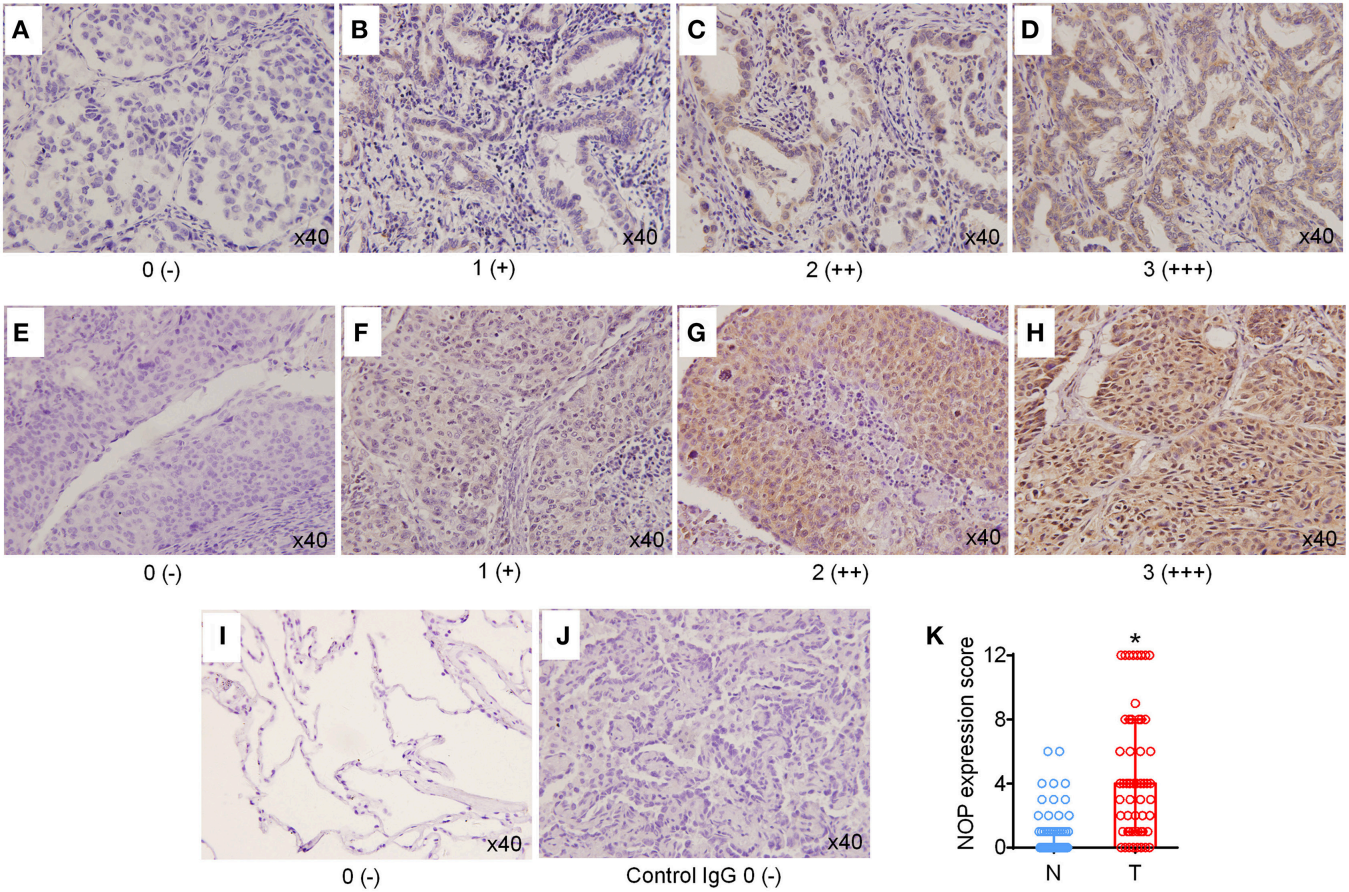
High expression levels of NOP in NSCLC tissues. **(A–D)** Representative immunohistochemical (IHC) staining of NOP in lung adenocarcinoma. Scores 0, 1, 2, and 3 represent negative (–), weak positive (+), moderate positive (++), and strong positive (+++) expression, respectively. **(E–H)** Typical IHC staining of NOP in lung squamous cell carcinoma. Scores 0, 1, 2, and 3 represent negative (–), weak positive (+), moderate positive (++), and strong positive (+++) expression, respectively. **(I)** Representative NOP staining in para-cancer normal lung tissue. **(J)** NOP staining with rabbit IgG Isotype control antibody. No non-specific background signal was detected. **(K)** Comparison of NOP expression score between tumor tissue (T) and the corresponding adjacent normal lung tissues (N). ^*^P < 0.05 from Mann-Whitney test.

**Figure 2:**
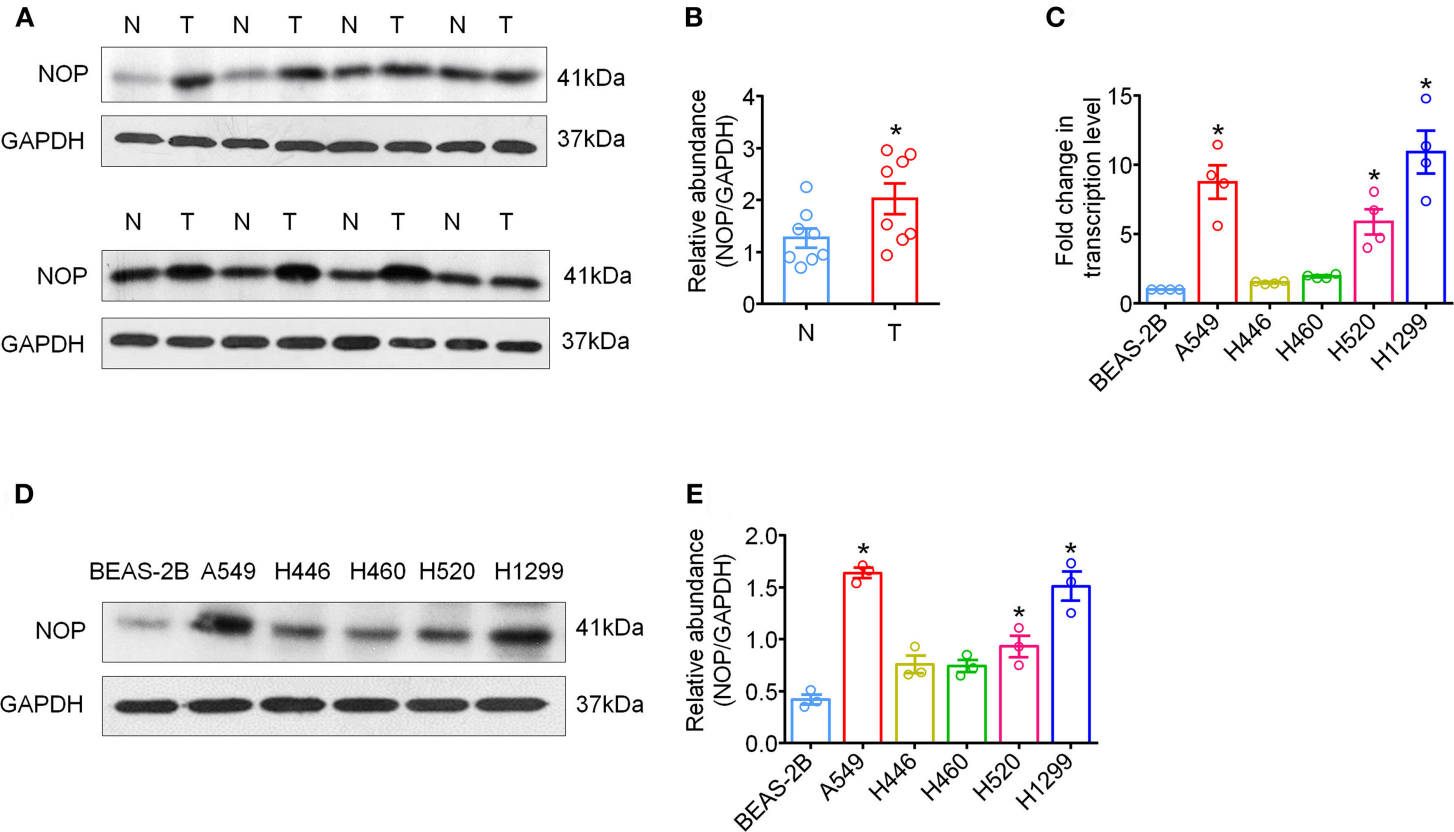
High expression levels of NOP in fresh tumor tissues and human NSCLC cell lines. **(A)** The global proteins of eight surgically removed tumor tissues (T) and the adjacent normal lung tissues (N) were extracted and the expression of NOP was assayed via western-blot, with GAPDH as an internal control. **(B)** Quantification of NOP expression via western-blot from fresh tumor tissues. ^*^P < 0.05 from paired *t*-test. **(C)** mRNA expression of NOP in human bronchial epithelial cell line and NSCLC cell lines with different histology. ^*^P < 0.05 compared with BEAS-2B from one-way ANOVA with Bonferroni's multiple comparisons test. **(D)** NOP expression in human bronchial epithelial cell line and NSCLC cell lines by western blot. **(E)** Quantification of NOP expression via western-blot from cell lines. ^*^P < 0.05 compared with BEAS-2B from one-way ANOVA with Bonferroni's multiple comparisons test.

Immunohistochemical staining was first performed to determine the expression levels of NOP in 129 cases of NSCLC tissues and 60 paired para-cancer tissues. The histological subtypes of these samples were SCC, (41 cases) and ADC (88 cases). NOP immunostaining was primarily visible in the plasma membrane and cytoplasm of cancer cells (Figures 1A–J). Approximately 44.2 % of all the examined cases in each subtype of lung carcinoma expressed high levels of NOP, with SCC (36.6%) and ADC (47.7%). When compared with the corresponding adjacent normal lung tissues, the cancer tissues showed a more intense staining and thus had a significantly higher expression score [4 (2/8) vs. 0 (0/1), *P* < 0.0001, Figure 1K]. To further validate these findings, eight paired tumor tissues and matched paired para-carcinoma samples with pathological diagnoses of adenocarcinoma or squamous cell carcinoma were used to perform the western blot assay and a consistent trend could be observed in accordance with the data from immunohistochemical staining (Figures 2A,B).

Next, we examined variations in NOP mRNA expression in human lung ADC cell lines (A549, H1299), human small cell lung cancer cell line (H446), human lung large cell carcinoma cell line (H460), human SCC cell line (H520), and normal lung bronchus cell line (BEAS-2B) by qRT-PCR and in NOP protein levels by western blot. A similar increase in NOP expression levels was observed in all histological subtypes of lung cancer cell lines and markedly in A549, H1299 and H520 cell lines (Figures 2C–E). However, the normal lung cell line, BEAS-2B, showed a weaker immunoblot reaction.

### NOP Expression and Clinicopathological Characteristics

The correlation between NOP expression levels on cancer cells and patients' clinicopathological characteristics are presented in Table 1. Elevated NOP expression was significantly associated with increased clinical TNM stage (*P* = 0.014), presence of lymph node invasion [Negative: 4 (2/8) vs. Positive: 5 (3/9), (*P* < 0.001)], and higher SUVmax [Median 9.5, patients were divided into < 9.5 group and ≥ 9.5 group, (*P* = 0.011)] suggesting a critical role of NOP in lung tumor growth and progression. However, no statistical relationship was found between NOP expression levels and opioid requirement (*P* = 0.073) as well as other clinical data such as age, gender, smoking status, histological type, and tumor size (all *P* > 0.05).

**Table 1:** Association between NOP expression level and clinicopathologic variables in patients with NSCLC.

	**Cases *n* (%)**	**NOP expression**			
		**Low**	**High**	***P*-value**	***X^**2**^***
**AGE (YEARS)**					
<60	50 (39)	24	26	0.155	2.021
≥60	79 (61)	48	31		
**GENDER**					
Male	74 (57)	42	32	0.802	0.063
Female	55 (43)	30	25		
**SMOKING STATUS**					
Never smoke	61 (47)	33	28	0.710	0.138
Smoke	68 (53)	39	29		
**HISTOLOGICAL TYPE**					
SCC	41 (32)	26	15	0.235	1.408
ADC	88 (68)	46	42		
**TNM STAGE**					
I	32 (25)	24	8	0.014	10.679
II	29 (22)	19	10		
III	63 (49)	27	36		
IV	5 (4)	2	3		
**TUMOR SIZE**					
≤3 cm	79 (61)	49	30	0.074	3.189
>3 cm	50 (39)	23	27		
**LYMPHATIC INVASION**					
Negative	47 (36)	32	15	0.034	4.515
Positive	82 (64)	40	42		
**SUVmax**					
<9.5	66 (51)	44	22	0.011	6.454
≥9.5	63 (49)	28	35		
**OPIOID REQUIREMENT**					
OME<5 mg/day	96 (74)	58	38	0.073	3.224
OME≥5 mg/day	33 (26)	14	19		

*NOP, nociceptin receptor; SCC, Squamous cell carcinoma; ADC, Adenocarcinoma; SUVmax, the maximum standardized uptake value; OME, oral morphine equivalents. Person chi-square test were used to analyze the data*.

### NOP Expression and Postoperative Prognosis

The median follow-up time was 43 months (ranging from 4 to 89 months). At the end of the follow- up, there were 40 deaths and 89 survivals. The 1-, 3-, and 5-years OS rates of all patients were 93.8, 74.7, and 63.1% respectively, and the corresponding PFS rate was 73.6, 49.3, and 43.1% respectively. Univariate analysis indicated that the OS was significantly associated with smoking status (*P* = 0.033), TNM status (*P* = 0.003), tumor size (*P* < 0.0001), lymphatic invasion (*P* = 0.017), SUVmax (*P* < 0.0001), opioid requirement (*P* = 0.010) and NOP expression (*P* < 0.0001), whereas PFS was directly influenced by TNM stage (*P* = 0.002), tumor size (*P* < 0.0001), lymphatic invasion (*P* = 0.002), SUVmax (*P* < 0.0001) and NOP expression (*P* < 0.0001). Multivariate analysis demonstrated that NOP expression levels and SUVmax were independent prognostic factors for OS, and tumor size and NOP expression were independent predictive factors for PFS (all *P* < 0.05, Tables 2, 3).

**Table 2:** Univariate and multivariate analysis of clinicopathologic factors in NSCLC patients with respect to OS.

	**Cases *n* (%)**	**Univariate analysis**		**Multivariate analysis**	
		**Survival time (months)**	***P*-value**	**HR (95%CI)**	***P*-value**
**AGE (YEARS)**					
<60	50 (39)	69.62 (4.40)	0.549		
≥60	79 (61)	62.72 (3.49)			
**GENDER**					
Male	74 (57)	63.43 (3.92)	0.177		
Female	55 (43)	66.22 (3.62)			
**SMOKING STATUS**					
Never smoke	61 (47)	68.13 (3.19)	0.033	1.156 (0.723–3.179)	0.271
Smoke	68 (53)	60.71 (4.18)			
**HISTOLOGICAL TYPE**					
SCC	41 (32)	58.64 (4.88)	0.164		
ADC	88 (68)	69.57 (3.46)			
**TNM STAGE**					
I-II	61 (47)	74.80 (3.47)	0.003	1.292 (0.533–3.133)	0.571
III-IV	68 (53)	55.67 (3.81)			
**TUMOR SIZE**					
≤3 cm	79 (61)	74.40 (3.18)	<0.0001	1.569 (0.713–3.453)	0.263
>3 cm	50 (39)	49.54 (4.60)			
**LYMPHATIC INVASION**					
Negative	47 (36)	74.54 (3.90)	0.017	1.743 (0.698–4.351)	0.234
Positive	82 (64)	59.16 (3.68)			
**SUVmax**					
<9.5	66 (51)	71.14 (3.02)	<0.0001	2.790 (1.196–6.504)	0.018
≥9.5	63 (49)	50.55 (3.82)			
**NOP EXPRESSION**					
Low	72 (56)	75.37 (3.33)	<0.0001	2.091 (1.035–4.227)	0.040
High	57 (44)	51.90 (4.05)			
**OPIOID REQUIREMENT**					
OME<5 mg/day	96 (74)	73.24 (3.15)	0.010	1.851 (0.732–4.744)	0.209
OME≥5 mg/day	33 (26)	57.25 (4.21)			

*SCC, Squamous cell carcinoma; ADC, Adenocarcinoma; SUVmax, the maximum standardized uptake value; NOP, nociceptin receptor; OME, oral morphine equivalents. Overall Survival (OS) time were presented as mean (SEM) and were determined by the Kaplan-Meier method with log-rank test. Multivariable analysis of the independent factors was performed by the Cox proportional hazard model*.

**Table 3:** Univariate and multivariate analysis of clinicopathologic factors in NSCLC patients with respect to PFS.

	**Cases *n* (%)**	**Univariate analysis**		**Multivariate analysis**	
		**Survival time (months)**	***P*-value**	**HR (95%CI)**	***P*-value**
**AGE (YEARS)**					
<60	50 (39)	45.56 (5.45)	0.962		
≥60	79 (61)	43.94 (4.04)			
**GENDER**					
Male	74 (57)	44.64 (4.37)	0.801		
Female	55 (43)	41.02 (3.69)			
**SMOKING STATUS**					
Never smoke	61 (47)	43.78 (3.46)	0.233		
Smoke	68 (53)	42.89 (4.49)			
**HISTOLOGICAL TYPE**					
SCC	41 (32)	44.47 (5.52)	0.711		
ADC	88 (68)	45.65 (4.11)			
**TNM STAGE**					
I-II	61 (47)	54.06 (4.55)	0.002	1.122 (0.563-2.237)	0.744
III-IV	68 (53)	31.47 (3.14)			
**TUMOR SIZE**					
≤3 cm	79 (61)	54.45 (4.08)	<0.0001	2.216 (1.289-3.808)	0.004
>3 cm	50 (39)	25.15 (3.26)			
**LYMPHATIC INVASION**					
Negative	47 (36)	56.05 (5.30)	0.002	1.665 (0.814-3.404)	0.163
Positive	82 (64)	38.26 (3.86)			
**SUVmax**					
<9.5	66 (51)	56.22 (4.39)	<0.0001	1.682 (0.945-2.995)	0.077
≥9.5	63 (49)	28.62 (3.16)			
**NOP EXPRESSION**					
Low	72 (56)	56.31 (4.39)	<0.0001	2.291 (1.394-3.767)	0.001
High	57 (44)	28.81 (3.56)			
**OPIOID REQUIREMENT**					
OME <5 mg/day	96(74)	46.55 (4.88)	0.833		
OME≥5 mg/day	33(26)	44.32 (4.16)			

*SCC, Squamous cell carcinoma; ADC, Adenocarcinoma; SUVmax, the maximum standardized uptake value; NOP, nociceptin receptor; OME, oral morphine equivalents. Progression free survival (PFS) time were presented as mean (SEM) and were determined by the Kaplan-Meier method with log-rank test. Multivariable analysis of the independent factors was performed by the Cox proportional hazard model*.

**Figure 3:**
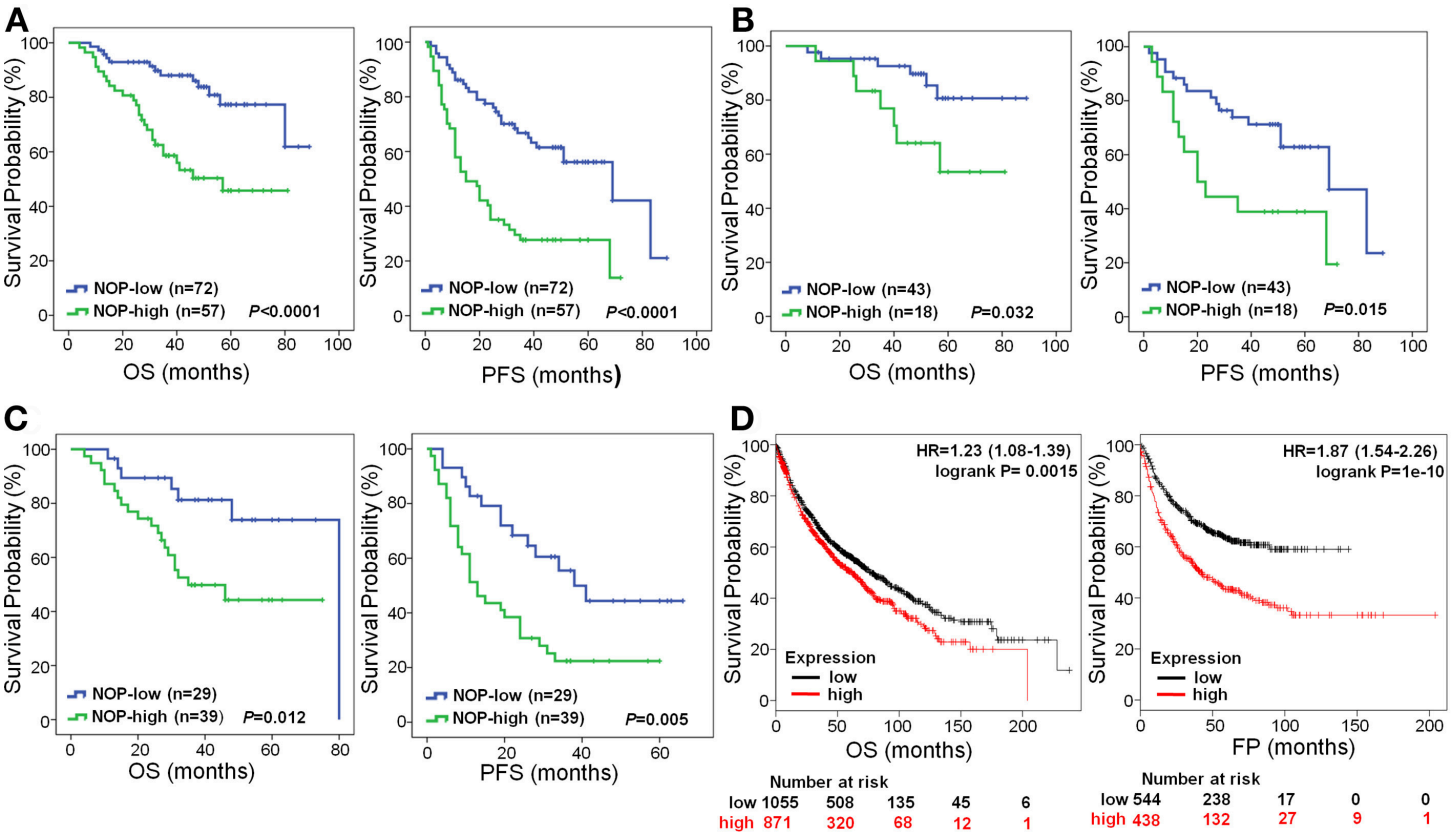
Survival curves of NSCLC patients according to NOP levels expressed in cancer. **(A)** All the enrolled patients with high NOP level were significantly associated with poor OS and PFS (both *P* < 0.001). **(B)** Reduced OS (*P* = 0.032) and PFS (*P* = 0.015) in patients with TNM stage I-II and high level of NOP. **(C)** Significantly poor OS (*P* = 0.012) and PFS (P = 0.005) in patients with TNM stage III-IV and expressing high level of NOP. **(D)** High level of NOP mRNA indicates a diminished OS (*P* = 0.0015) and first progression (FP, *P* < 0.0001) time in NSCLC patients, by Kaplan-Meier Plotter online tool. Kaplan-Meier method was applied to determine OS and PFS with log-rank test.

The 1, 3, and 5-year OS rate of patients with low NOP expression was 97.2, 88.0, and 77.3%, respectively. However, that of patients with high NOP expression, decreased to 89.5, 58.6, and 45.8%, respectively (*P* < 0.0001, Figure 3A). The 1, 3, and 5-year PFS rate for patients with low NOP expression was 86.1, 66.8, and 56.1%, respectively, and 57.9, 27.7, and 27.7% for patients with high NOP expression (*P* < 0.0001, Figure 3A). These data illustrate that NOP overexpression is related to a poor postoperative prognosis. Since NOP expression was linked with TNM stages, we further performed survival analysis in patients with subtype clinical stage I -II and III-IV. For stage I-II subgroup, patients showing high levels of NOP had shorter OS and PFS than those showing low NOP levels (both *P* < 0.05, Figure 3B). A similar trend could also be observed in patients of stage III-IV, where high expression of NOP led to reduced OS and PFS (both *P* < 0.05, Figure 3C). Since for advanced stage NSCLC patients, epidermal growth factor receptor (EGFR) mutation status may play a role in postoperative outcome, we further compared the EGFR sensitive mutation ratio in NOP high expression patients and NOP low expression patients with stage III-IV and found no statistical difference (53.8 vs. 48.3%, respectively, *P* = 0.649).

To further verify the prognostic role of NOP in NSCLC patients, the online tool of Kaplan-Meier Plotter was used to determine mRNA expression levels of NOP from multiple databases (including GEO, EGA and TCGA). As shown in Figure 3D, high expression levels of NOP indicated a lower OS in 1926 patients (HR:1.23, 95% CI: 1.08–1.39, *P* = 0.0015) and a decreased first progression (FP) time in 982 patients (HR:1.87, 95% CI: 1.54–2.26, *P* < 0.0001) with histological diagnosis of lung ADC and SCC.

### NOP Activation Promotes the Proliferation and Invasion of A549 Cells via PI3K/Akt Signaling Pathway

**Figure 4:**
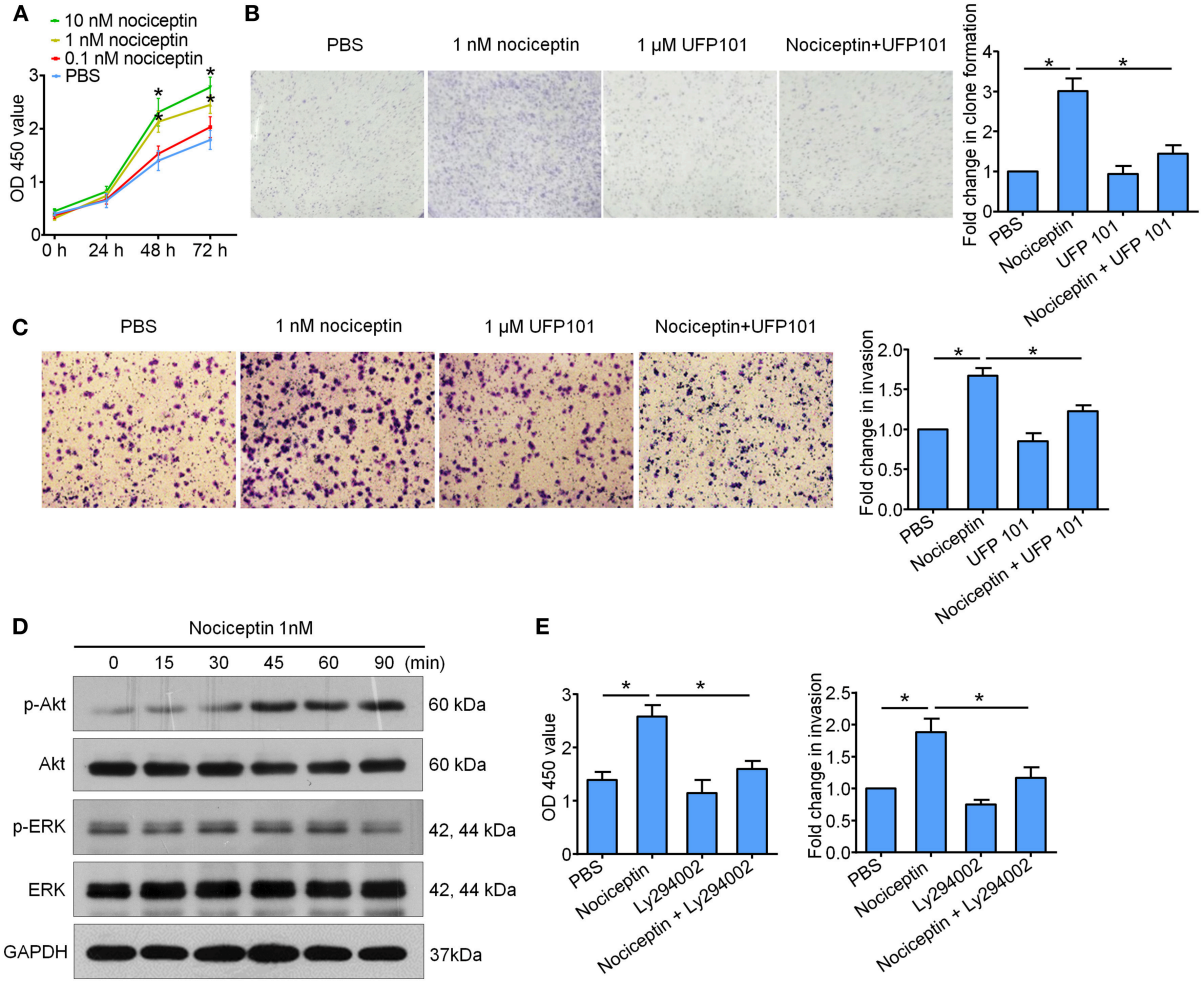
Nociceptin promotes the proliferation and invasion of A549 cells through NOP with the activation of PI3K/Akt signaling pathway. **(A)** Nociceptin of 1 nM and 10 nM could increase the proliferation of A549 cells by CCK8 test. ^*^P < 0.05 compared with PBS from two-way ANOVA with Bonferroni's multiple comparisons test. **(B)** 1 nM nociception could enhance the clone forming ability of A549 cells which would be diminished by co-incubation with 1 μM UFP 101. Representative pictures were shown on the left, and the fold change were shown on the right. **(C)** Nociceptin of 1 nM might boost the ability of invasion of A549 cells, which would be significantly impaired by co-incubation with 1 μM UFP 101. Representative pictures were shown on the left, and the quantification was presented on the right. **(D)** A549 cells were treated with 1nM Nociceptin for indicated time points before subjected to the examination of PI3K/Akt and ERK signaling pathway. Significant activation of Akt pathway but not ERK pathway was detected. **(E)** 10 μM Ly294002 could inhibit the enhancement of proliferation and invasion ability of A549 cells caused by 1 nM Nociceptin. For figure **(B,C,E)**, ^*^P < 0.05 from one-way ANOVA with Bonferroni's multiple comparisons test.

*In vitro* experiments with A549 cell line high expressing NOP were performed to assess NOP activation on tumor aggressive features. We firstly used CCK-8 and 2D clone formation assays to detect the change of cell proliferation. As is shown in Figures 4A,B, NOP agonist nociceptin could dose dependently enhance the proliferation ability of A549 cells with remarkable effect of 1 nM and 10 nM and these could be diminished by co-incubation with NOP antagonist UFP 101 (1 μM). In addition, 1 nM nociceptin may boost the ability of invasion of A549 cells, which would be significantly impaired by the application of 1 μM UFP 101 (Figure 4C), since opioids related growth-promoting effects were mainly reported to be mediated through extracellular-signal regulated kinase (ERK) and PI3K/Akt signaling pathways (21). We screened these two pathways in A549 cells treated with 1 nM nociception at different time points and found that the p-Akt level but not the p-ERK level were significantly increased after the treatment (Figure 4D). In functional experiments, PI3K/Akt pathway inhibitor Ly294002 (10 μM) could impair the enhancement of proliferation and invasion ability of A549 cells caused by 1 nM nociception (Figure 4E).

## Discussion

In the present study, we investigated NOP expression in cancer patients beyond the neural and immune systems. Results showed that NOP was highly expressed in the cancer tissues and human cancer cell lines when compared to normal lung tissue and human bronchus cell line. Moreover, we demonstrated that NOP expression on cancer cells was closely associated with patients' clinicopathological data reflecting the malignancy. NOP staining levels in cancerous tissue were the independent poor prognostic factor for NSCLC patients receiving pulmonary resection, which was further verified by determining mRNA expression levels through the online dataset.

Retrospective studies have shown that opioid requirement and administration dosage is inversely related to poor outcomes of lung cancer patients receiving perioperative analgesia or cancer pain relief therapy (22, 23). Thus, it is essential to understand the expression of different opioid receptors in tumor microenvironments and their interaction with endogenous and exogenous opioids. As the classic opioid receptors, the roles of MOR, DOR, and KOR in NSCLC have been established and depicted widely (4, 5, 7, 24). Meanwhile, one non-classical opioid receptor, the opioid growth factor receptor (OGFR), which is known as the receptor of endogenous opioid peptide [Met5]-enkephalin, was also shown to be expressed in lung cancer tissues and cancer cell lines involved in morphine induced suppression of lung cancer proliferation (25). Aside from the direct interaction of opioids and their receptors expressed in tumors, the immunological effects of opioids were also investigated, mainly targeting MOR on immunocytes such as natural killer (NK) cells and T lymphocytes (26, 27). However, one latest research reported the absent expression of classical MOR, DOR and KOR in human blood samples which contain various types of immune cells, while NOP were detected in all samples (28). This implied NOP may be more crucial than classic opioid receptors in the direct modulation of immune function. Based on these findings, the expression and potential role of NOP in cancer and tumor immune microenvironment is in need to be clarified.

Previously, NOP was reported to be functionally expressed in tumor cell lines of neuroblastoma and glioblastoma. However, limited research explored NOP expression in other cancer cell lines and further in cancer patients. Stamer and colleagues assayed NOP and nociceptin precursor pre-pronociceptin expression by qRT-PCR in end-stage cancer patients, including patients of the gastrointestinal tract, lung, breast, and cervix cancer (16). Results showed that expression of NOP components in the peripheral blood of cancer patients was significantly higher than that in healthy controls. Moreover, no correlation was found between pre-pronociceptin or NOP expression levels and pain intensity in cancer related studies of NOP (16, 29, 30). In the present study, we also found that NOP expression was not related to patients' opioid requirements. These all indicated a distinct role of NOP in tumor microenvironment, apart from pain signaling. Here, we detected the NOP expression directly in tumor cell lines and cancer samples instead of plasma immune cells. We demonstrated high expression levels of NOP in NSCLC cell lines and in cancer cells of cancer tissues and their correlation with cancer progression, which illustrated the role of NOP components in tumor progression.

Based on the finding that NOP was a poor prognostic factor for NSCLC patients, the *in vitro* experiments in this study further verified that NOP activation by its endogenous ligand - nociceptin in a low dose could promote the proliferative and invasive function of A549 cells. Recently, emerging studies have reported that NOP antagonist exerted anti-proliferation or anti-metastasis effects in osteosarcoma, ovarian cancer and pancreatic cancer cell lines (31–33). These all raised the possible cancer promoting role of NOP. Although one research showed that nociception could block the lipopolysaccharide induced proliferation and migration in human glioblastoma U87 cells (34), it used a much higher dose of nociceptin of 1 μM. Also, GPCR activation has already been proved to play a distinct role in tumor biology depending not only on target cell lines but also on the investigation system (*in vivo* or *in vitro*) (35, 36). Principally, growth-promoting effects were mediated through ERK and PI3K/Akt signaling pathways, whereas death-promoting effects were ascribed to increment of Fas expression, p53 stabilization, and inhibition of nuclear factor-κB (21). Stimulation of NOP by agonist was demonstrated to induce activation of all three mitogen-activated protein kinase (MAPK) cassettes. Specially, two groups have reported increased ERK 1/2 phosphorylation levels in heterologous expression systems after NOP activation (37, 38). In astrocyte, the participation of downstream pathways of PI3K/AKT kinase system in NOP simulation has also been proved (39). In the present study, we checked the dynamic change of ERK and Akt pathways and found that PI3K/Akt pathways involved in NOP activation induced tumor progression. Thus, PI3K/Akt inhibitor might be used to treat NSCLC patients with high expression level of NOP.

There are several limitations to this study. First, we only took one human normal lung cell line as control in the comparison of NOP expression in various cell lines. Second, we did not detect the expression of nociceptin or its precursor protein in cancer tissues or in the plasma of enrolled patients. In addition, the population size of patients in advanced stage is small. It has been reported that in inflammation models immune cells like neutrophils could migrate and secrete nociceptin, which would couple NOP in nerve cells and trigger downstream pathways to release proinflammatory cytokines, such as IL-6 and IL-1 β (15), which are generally considered to be tumor promoters (40, 41). Interestingly, in the tumor immune microenvironment, whether infiltrated immune cells could increasingly produce nociceptin and further activate NOP signaling pathways in surrounding cancer cells is worthy of investigation.

In summary, we proved that NOP is overexpressed in NSCLC and is inversely correlated with patient's postoperative survival. Activation of NOP on tumor cells can promote their proliferative and invasive characteristics. All these results revealed a potential diagnostic and therapeutic role of NOP in NSCLC patients.

## Ethics Statement

This study was approved by the Ethical Committee of Tianjin Medical University Cancer Institute and Hospital (TMUCIH), and a written, informed consent was obtained from each participant or their legal custodian, in accordance with the Declaration of Helsinki.

## Author Contributions

XR, HZ, and KW conceived and designed the experiments. KW, YZ, YY, JW, and BL performed the experiments and wrote the manuscript. FW and KW analyzed the data. HZ and XR revised the manuscript.

### Conflict of Interest Statement

The authors declare that the research was conducted in the absence of any commercial or financial relationships that could be construed as a potential conflict of interest.

## References

[1] Hirsch FR Scagliotti GV Mulshine JL Kwon R Curran WJ Jr. Wu YL. Lung cancer: current therapies and new targeted treatments. Lancet. (2017) 389:299–311. 10.1016/s0140-6736(16)30958-8 27574741

[2] Siegel RL Miller KD Jemal A Cancer statistics, 2017. CA Cancer J Clin. (2017) 67:7–30. 10.3322/caac.21387 28055103

[3] Schug SA Chandrasena C. Pain management of the cancer patient. Expert Opin Pharmacother. (2015) 16:5–15. 10.1517/14656566.2015.980723 25479712

[4] Schreiber G Campa MJ Prabhakar S O'Briant K Bepler G Patz EF Jr. Molecular characterization of the human delta opioid receptor in lung cancer. Anticancer Res. (1998) 18:1787–792. 9673405

[5] Singleton PA Mirzapoiazova T Hasina R Salgia R Moss J. Increased mu-opioid receptor expression in metastatic lung cancer. Br J Anaesth. (2014) 113 (Suppl 1):i103–108. 10.1093/bja/aeu165 24920011 PMC4111280

[6] Fujioka N Nguyen J Chen C Li Y Pasrija T Niehans G. Morphine-induced epidermal growth factor pathway activation in non-small cell lung cancer. Anesth Analg. (2011) 113:1353–64. 10.1213/ANE.0b013e318232b35a 22003224 PMC3725599

[7] Kuzumaki N Suzuki A Narita M Hosoya T Nagasawa A Imai S. (2012). Effect of kappa-opioid receptor agonist on the growth of non-small cell lung cancer (NSCLC) cells. Br J Cancer. (2012) 106:1148–52. 10.1038/bjc.2011.574 22343623 PMC3304401

[8] Civelli O Reinscheid RK Zhang Y Wang Z Fredriksson R Schioth HB. G protein-coupled receptor deorphanizations. Annu Rev Pharmacol Toxicol. (2013) 53:127–46. 10.1146/annurev-pharmtox-010611-134548 23020293 PMC5828024

[9] Calo G Guerrini R Rizzi A Salvadori S Regoli D. Pharmacology of nociceptin and its receptor: a novel therapeutic target. Br J Pharmacol. (2000) 129:1261–83. 10.1038/sj.bjp.0703219 10742280 PMC1571975

[10] Tehan BG Bortolato A Blaney FE Weir MP Mason JS. Unifying family A GPCR theories of activation. Pharmacol Ther. (2014) 143:51–60. 10.1016/j.pharmthera.2014.02.004 24561131

[11] Meunier JC Mollereau C Toll L Suaudeau C Moisand C Alvinerie P. Isolation and structure of the endogenous agonist of opioid receptor-like ORL1 receptor. Nature. (1995) 377: 532–35. 10.1038/377532a0 7566152

[12] Chung S Pohl S Zeng J Civelli O Reinscheid RK. Endogenous orphanin FQ/nociceptin is involved in the development of morphine tolerance. J Pharmacol Exp Ther. (2006) 318:262–7. 10.1124/jpet.106.103960 16595734

[13] Marti M Mela F Budri M Volta M Malfacini D Molinari S. Acute and chronic antiparkinsonian effects of the novel nociceptin/orphanin FQ receptor antagonist NiK-21273 in comparison with SB-612111. Br J Pharmacol. (2013) 168:863–79. 10.1111/j.1476-5381.2012.02219.x 22994368 PMC3631376

[14] Toll L Bruchas MR Calo G Cox BM Zaveri NT. Nociceptin/Orphanin FQ receptor structure, signaling, ligands, functions, and interactions with opioid systems. Pharmacol Rev. (2016) 68: 419–57. 10.1124/pr.114.009209 26956246 PMC4813427

[15] Fiset ME Gilbert C Poubelle PE Pouliot M. Human neutrophils as a source of nociceptin: a novel link between pain and inflammation. Biochemistry. (2003) 42:10498–505. 10.1021/bi0300635 12950177 PMC2881299

[16] Stamer UM Book M Comos C Zhang L Nauck F Stuber F. Expression of the nociceptin precursor and nociceptin receptor is modulated in cancer and septic patients. Br J Anaesth. (2011) 106:566–72. 10.1093/bja/aer007 21324928

[17] Asamura H Chansky K Crowley J Goldstraw P Rusch VW Vansteenkiste JF. The International Association for the study of lung cancer lung cancer staging project: proposals for the revision of the N descriptors in the forthcoming 8th Edition of the TNM classification for lung cancer. J Thorac Oncol. (2015) 10: 1675–84. 10.1097/JTO.0000000000000678 26709477

[18] Gammaitoni AR Fine P Alvarez N McPherson ML Bergmark S. Clinical application of opioid equianalgesic data. Clin J Pain. (2003) 19:286–97. 12966254 10.1097/00002508-200309000-00002

[19] Zylla D Kuskowski MA Gupta K Gupta P. Association of opioid requirement and cancer pain with survival in advanced non-small cell lung cancer. Br J Anaesth. (2014) 113:i109–16. 10.1093/bja/aeu351 25303989 PMC6223789

[20] Wang K Wang J Wei F Zhao N Yang F Ren X. Expression of TLR4 in non-small cell lung cancer is associated with PD-L1 and poor prognosis in patients receiving pulmonectomy. Front Immunol. (2017) 8:456. 10.3389/fimmu.2017.00456 28484456 PMC5399072

[21] Tegeder I Geisslinger G. Opioids as modulators of cell death and survival–unraveling mechanisms and revealing new indications. Pharmacol Rev. (2004) 56:351–369. 10.1124/pr.56.3.2 15317908

[22] Maher DP Wong W White PF McKenna R Jr. Rosner H Shamloo B. Association of increased postoperative opioid administration with non-small-cell lung cancer recurrence: a retrospective analysis. Br J Anaesth. (2014) 113 (Suppl 1):i88–94. 10.1093/bja/aeu192 25009195

[23] Cata JP Keerty V Keerty D Feng L Norman PH Gottumukkala V. A retrospective analysis of the effect of intraoperative opioid dose on cancer recurrence after non-small cell lung cancer resection. Cancer Med. (2014) 3:900–8. 10.1002/cam4.236 24692226 PMC4303157

[24] Mathew B Lennon FE Siegler J Mirzapoiazova T Mambetsariev N Sammani S. The novel role of the mu opioid receptor in lung cancer progression: a laboratory investigation. Anesth Analg. (2011) 112:558–67. 10.1213/ANE.0b013e31820568af 21156980 PMC4327979

[25] Kim JY Ahn HJ Kim JK Kim J Lee SH Chae HB Morphine suppresses lung cancer cell proliferation through the interaction with opioid growth factor receptor: an *in vitro* and human lung tissue study. Anesth Analg. (2016) 23:1429–36. 10.1213/ane.0000000000001293 27167686

[26] Snyder GL Greenberg S. Effect of anaesthetic technique and other perioperative factors on cancer recurrence. Br J Anaesth. (2010) 105: 106–15. 10.1093/bja/aeq164 20627881

[27] Sacerdote P. Opioids and the immune system. Palliat Med. (2006) 20 (Suppl 1):s9–15. 16764216

[28] Al-Hashimi M McDonald J Thompson JP Lambert DG. Evidence for nociceptin/orphanin FQ (NOP) but not micro (MOP), delta (DOP) or kappa (KOP) opioid receptor mRNA in whole human blood. Br J Anaesth. (2016) 116: 423–9. 10.1093/bja/aev540 26865135

[29] Szalay F Hantos MB Horvath A Lakatos PL Folhoffer A Dunkel K. Increased nociceptin/orphanin FQ plasma levels in hepatocellular carcinoma. World J Gastroenterol. (2004) 10:42–5. 10.3748/wjg.v10.i1.42 14695766 PMC4717075

[30] Horvath A Folhoffer A Lakatos PL Halosz J Illyes G Schaff Z. Rising plasma nociceptin level during development of HCC: a case report. World J Gastroenterol. (2004) 10:152–154. 10.3748/wjg.v10.i1.152 14695788 PMC4717069

[31] Zheng CJ Yang LL Liu J Zhong L. JTC-801 exerts anti-proliferative effects in human osteosarcoma cells by inducing apoptosis. J Recept Signal Transduct Res. (2018) 38:133–40. 10.1080/10799893.2018.1436561 29447541

[32] Song X Zhu S Xie Y Liu J Sun L Zeng D. JTC801 Induces pH-dependent death specifically in cancer cells and slows growth of tumors in mice. Gastroenterology. (2018) 154:1480–93. 10.1053/j.gastro.2017.12.004 29248440 PMC5880694

[33] Li JX Bi YP Wang J Yang X Tian YF Sun ZF. JTC-801 inhibits the proliferation and metastasis of ovarian cancer cell SKOV3 through inhibition of the PI3K - AKT signaling pathway. Pharmazie. (2018) 73:283–87. 10.1691/ph.2018.7326 29724295

[34] Bedini A Baiula M Vincelli G Formaggio F Lombardi S Caprini M. Nociceptin/orphanin FQ antagonizes lipopolysaccharide-stimulated proliferation, migration and inflammatory signaling in human glioblastoma U87 cells. Biochem Pharmacol. (2017) 140:89–104. 10.1016/j.bcp.2017.05.021 28583844

[35] Singhal PC Sharma P Sanwal V Prasad A Kapasi A Ranjan R. Morphine modulates proliferation of kidney fibroblasts. Kidney Int. (1998) 53:350–7. 10.1046/j.1523-1755.1998.00758.x 9461094

[36] Ishikawa M Tanno K Kamo A Takayanagi Y Sasaki K. Enhancement of tumor growth by morphine and its possible mechanism in mice. Biol Pharm Bull. (1993) 16:762–6 8220322 10.1248/bpb.16.762

[37] Lou LG Zhang Z Ma L Pei G. Nociceptin/orphanin FQ activates mitogen-activated protein kinase in Chinese hamster ovary cells expressing opioid receptor-like receptor. J Neurochem. (1998) 70:1316–22. 9489755 10.1046/j.1471-4159.1998.70031316.x

[38] Zhang NR Planer W Siuda ER Zhao HC Stickler L Chang SD. Serine 363 is required for nociceptin/orphanin FQ opioid receptor (NOPR) desensitization, internalization, and arrestin signaling. J Biol Chem. (2012) 287:42019–30. 10.1074/jbc.M112.405696 23086955 PMC3516748

[39] Meyer LC Paisley CE Mohamed E Bigbee JW Kordula T Richard H. Novel role of the nociceptin system as a regulator of glutamate transporter expression in developing astrocytes. Glia. (2017) 65:2003–23. 10.1002/glia.23210 28906039 PMC5766282

[40] Grivennikov S Karin E Terzic J Mucida D Yu GY Vallabhapurapu S. IL-6 and Stat3 are required for survival of intestinal epithelial cells and development of colitis-associated cancer. Cancer Cell. (2009) 15:103–13. 10.1016/j.ccr.2009.01.001 19185845 PMC2667107

[41] Wang L Zhang LF Wu J Xu SJ Xu YY Li D. IL-1beta-mediated repression of microRNA-101 is crucial for inflammation-promoted lung tumorigenesis. Cancer Res. (2014) 74:4720–30. 10.1158/0008-5472.can-14-0960 24958470

